# Evaluation of Attention-Deficit Hyperactivity Disorder Risk Factors

**DOI:** 10.1155/2013/953103

**Published:** 2013-11-10

**Authors:** Javad Golmirzaei, Shole Namazi, Shahrokh Amiri, Shahram Zare, Najme Rastikerdar, Ali Akbar Hesam, Zahra Rahami, Fatemeh Ghasemian, Seyyed Shojaeddin Namazi, Abbas Paknahad, Forugh Mahmudi, Hamidreza Mahboobi, Tahereh Khorgoei, Bahareh Niknejad, Fatemeh Dehghani, Shima Asadi

**Affiliations:** ^1^Research Center for Behavioral and Neurosciences, Hormozgan University of Medical Sciences, Bandar Abbas, Iran; ^2^Clinical Psychiatry Research Center, Tabriz University of Medical Sciences, Tabriz, Iran; ^3^Department of Psychiatry, Razi Hospital, Elgoli Road, Tabriz, East Azerbaijan, Iran; ^4^Hormozgan University of Medical Sciences, Bandar Abbas, Iran; ^5^Department of Epidemiology, Islamic Azad University, Bandar Abbas, Iran; ^6^Student Research Committee, Hormozgan University of Medical Sciences, Bandar Abbas, Iran; ^7^Tropical and Infectious Disease Research Center, Hormozgan University of Medical Sciences, Bandar Abbas, Iran; ^8^Fertility and Infertility Research Center, Hormozgan University of Medical Sciences, Bandar Abbas, Iran

## Abstract

*Background*. Attention-deficit hyperactivity disorder (ADHD) is one of the most common psychiatric disorders among children. The aim of this study was to evaluate risk factors for ADHD in children. *Method*. In this case-control study, 404 children between 4 and 11 years old were selected by cluster sampling method from preschool children (208 patients as cases and 196 controls). All the participants were interviewed by a child and adolescent psychiatrist to survey risk factors of ADHD. *Results*. Among cases, 59.3% of children were boys and 38.4% were girls, which is different to that in control group with 40.7% boys and 61.6% girls. The chi-square showed statistically significance (*P* value < 0.0001). The other significant factors by chi-square were fathers' somatic or psychiatric disease (*P* value < 0.0001), history of trauma and accident during pregnancy (*P* value = 0.039), abortion proceeds (*P* value < 0.0001), unintended pregnancy (*P* value < 0.0001), and history of head trauma (*P* value < 0.0001). *Conclusions*. Findings of our study suggest that maternal and paternal adverse events were associated with ADHD symptoms, but breast feeding is a protective factor.

## 1. Introduction

Attention-deficit hyperactivity disorder (ADHD) is one of the most common behavioral and neurodevelopmental disorders which is characterized by hyperactivity, impulsivity, and inattention in children and adolescents [1]. Prevalence of ADHD among school-aged children in different studies varies between 5 and 12% [1, 2], whereas the prevalence of this disorder declines with increasing age [3]. It is estimated that this disorder persists into adulthood in 50% of afflicted children [4]. Children with ADHD are at increased risk of antisocial behavior, learning disabilities, drug abuse, impaired academic performance, impaired executive functions, communication disorders, speech problems, and comorbid psychiatric disorders [5, 6]. Although, the pathogenesis of ADHD is still unknown, primary and secondary factors are estimated to be implicated in ADHD pathogenesis. Primary roles are shaped in the cerebral cortex by catecholamine metabolism. Also, etiology of ADHD is attributed to genetic factors in about 80% [7]. The secondary roles are created by various environmental factors [8, 9]. Some of these factors, which are associated with ADHD, are pregnancy and birth related risk factors which are classified into three groups including prenatal, perinatal, and postnatal risk factors. Regarding prenatal risk factors, a large number of studies have shown that maternal exposure to alcohol, tobacco, and cocaine during pregnancy increases the risk of ADHD. On the other hand, some studies showed that prenatal viral infections are associated with increased risk of ADHD [10, 11]. Various studies have demonstrated that preeclampsia, maternal anemia, lower serum level of iron and iodine, and trauma to abdomen during pregnancy are associated with increased risk of ADHD development [10, 12]. Regarding perinatal risk factors, a number of risk factors such as prematurity, low birth weight, and breech delivery are estimated to be associated with increased risk of ADHD [13]. Postnatal risk factors include postnatal viral infections such as measles, varicella, and rubella increasing the risk of developing ADHD [13]. Additionally, several other factors such as breast-feeding, head injury in early childhood and adolescence, encephalitis, convulsion and endocrine disorder are estimated to be risk factors for development of ADHD [13, 14].

Although the association of breastfeeding and ADHD is not established, some studies have shown that ADHD is more common in children with lower rates of breast feeding [15]. The role of other factors such as unintended pregnancy, X-ray exposure, and previous abortion is not established [16]. In addition to the mentioned factors, several sociodemographic factors such as maternal education, family income, male gender, and maternal age at pregnancy are known to be predictive factors for developing ADHD [12, 13]. Some studies have shown that maternal emotional stress during pregnancy is related to increased risk of psychobehavioral disorders such as ADHD [17]. Prematurity, maternal alcohol consumption, and smoking are associated with ADHD symptoms [15, 16]. However, data on other risk factors are less well established. With respect to destructive complication of ADHD and its negative effects on psychosocial behavior of children with this disorder, we aimed to determine the ADHD risk factors among the school-aged children in Bandar Abbas.

## 2. Method

This case-control study was carried out among 404 children aged 4–11 years that were selected by cluster sampling in southern Iran in 2012. This study was approved by the ethics committee of Hormozgan University of Medical Sciences (HUMS). Informed consent was obtained from their parents or legal guardians. In this study, 935 preschool children were selected using cluster sampling and all of them were screened by Conners' parents and teachers rating scales. Out of them, 271 children were positive for ADHD according to Conners' questionnaire and those who were positive for ADHD (271) were interviewed by a child and adolescent psychiatrist and 208 children who met DSM-IV criteria for ADHD were enrolled in this study.

This questionnaire had 28 items with answers based on Likert scales. The total possible score was 84 and children with a parent or teacher questionnaire total score above 70 were considered as positive and referred to Ebne-Sina Hospital, the only behavioral and neuroscience center in Hormozgan Province.

All of the selected children were interviewed by a specialist in child and adolescent psychiatry. The cases were selected among patients who met the diagnostic criteria of DMS-IV for ADHD and controls were chosen among healthy children. The participants in each group were matched for age. Their parents were asked to answer the questionnaire including maternal and pregnancy related risk factors for ADHD such as the mother age at pregnancy, thyroid disease, the length of pregnancy, exposure to X-ray, infectious disease, eclampsia, abnormal uterine bleeding, abdominal trauma, medication intake, alcohol and tobacco consumption during pregnancy, and previous abortion, as well as neonatal and infantile related risk factors such as unwanted pregnancy, type of delivery, hyperbilirubinemia, phototherapy, blood exchange, low birth weight (less than 2.5 kg), feeding (breastfeeding or formula feeding), childhood asthma, dysentery, epilepsy, head trauma, and thyroid disease. In addition, parents' educational level, economic level, and presence of psychiatric disease in children and their parents were documented.

Data was analyzed using statistical procedures for social sciences (SPSS; version 19) by descriptive analysis such as mean, standard deviation and percentile frequency. Differences between case and control groups were analyzed by chi-square test. A value of *P* < 0.05 was considered significant.

## 3. Results

A total of 404 children met the enrollment criteria, including 208 children as case group and 196 as controls. The mean age of participants in case and control groups was 6.48 ± 1.95 and 5.77 ± 1.232 years, respectively. There was no significant difference between both groups (*P* > 0.05). 

Among ADHD children, 150 (59.3%) were boys and 58 (38.4%) were girls. Among controls, 103 (40.7%) and 93 (61.6%) were boys and girls, respectively. Chi-square demonstrated that ADHD was significantly higher among boys than girls (*P* < 0.0001, OR: 3, 95% CI: (1.34–6.635)).

The results showed that a history of trauma to abdomen during pregnancy was significantly higher among mothers who had children with ADHD. Maternal, pregnancy, neonatal risk factors for ADHD, and maternal and paternal history of psychiatric disorders are listed in Tables 1 and 2.

As shown in Table 3, among neonatal and childhood risk factors, childhood head trauma and epilepsy were associated with AHDH.

## 4. Discussion

This cross-sectional case-control study was performed to determine the risk factors of ADHD.

Symptoms of ADHD almost present in early childhood after 3 years of age and before 7. It may be continued to adulthood and affect the interpersonal impairments, academic performance, and work and family problems. Untimely diagnosis and treatment of this disorder can lead to cognitive and behavioral impairments. In recent years, numerous investigations have been done to identify the etiology of this disorder and the related factors.

In the current study, ADHD was diagnosed three times in male children compared to females. This finding was consistent with the results of other studies conducted by Kim et al. [18] and Cantwell [19].

There are converging lines of evidence regarding the role of genetic factors in developing ADHD [20]. Our findings showed that parental psychiatric disorder was significantly associated with ADHD in children. This finding was consistent with previous studies [21, 22]. Moreover, our results showed that ADHD was more frequent among those children of nonrelated parents compared to related parents. This could be due to the increased chance of distribution of genetic role and mutation when children are born to nonrelated parents.

Controversial findings have been reported about the role of thyroid hormones in ADHD [23, 24], while some lines of evidence revealed that ADHD is more frequent in patients with a generalized resistance to thyroid hormones compared to normal population [25]. Our finding showed that thyroid disease was not significantly higher among patients with ADHD compared to control children. Screening of ADHD children for thyroid disease is not recommended unless the symptoms of hypo/hyperthyroidism are observed [26]. 

Alcohol exposure during pregnancy is related to increased risk of neurodevelopmental and conductive disorders. This association is well documented [27]. Our results showed that alcohol and tobacco exposure during pregnancy would increase the risk of ADHD. These findings are consistent with the results of other authors [28–30]. 

There are few studies regarding the association of preeclampsia in pregnancy and ADHD in their children. Some studies have shown that pregnancy complications are associated with increased risk of offspring ADHD [10, 31]. However, Amiri reported that a history of preeclampsia and infections during pregnancy were not significantly higher among mothers of children with ADHD [12]. Our findings revealed that trauma to abdomen, previous abortion, unwanted pregnancy, preeclampsia, and cesarean section were significantly higher among patients with ADHD compared to control subjects. But X-ray exposure, infectious disease, dysentery, and vaginal bleeding at pregnancy were not associated with increased risk of ADHD. 

Various studies demonstrated that low birth weight among ADHD children was significantly higher than normal children [28, 32]. But in this study, low birth weight was not significantly higher in children with ADHD.

The results of our study demonstrated that prevalence of ADHD among formula-fed patients was significantly higher than breast-fed children. This finding is consistent with the results of other studies. Some lines of evidence demonstrated that fatty acid compositions of human breast milk such as docosahexaenoic acid and arachidonic acid have an important role in brain development [33, 34]. The results of our study may be due to the role of fatty acids in brain growth during neonatal and infancy stages [35]. In addition, a review by Mazza and colleagues reported that fatty acids are associated with other psychotic disorders [36]. 

Our results showed that epilepsy and trauma to head were significantly higher among ADHD children than control group. On the other hand, the difference of the frequency of childhood asthma, dysentery, and thyroid disease was not significant between both groups. Our result was consistent with a study conducted by Gerring and colleagues that reported that ADHD was more frequent among patients who presented with moderate and severe head injuries [37]. Furthermore, another finding that was consistent with our results was reported by Dunn and colleagues; they demonstrated that children with epilepsy are at increased risk of ADHD in the future [38]. It is estimated that about 20% of children with epilepsy may develop ADHD [39]. Recently, various studies examine the effect of methylphenidate on seizure frequency among patients with concomitant ADHD and epilepsy. These evidences showed that methylphenidate is an effective and safe medication for patients with both epilepsy and ADHD [39, 40].

Although in this study we tried to eliminate the overestimation or underestimation of ADHD by parents of children, whoever they are, a limitation in our study could be the probable recall bias by the parents. 

In conclusion, our study demonstrated that parental psychiatric disorders, previous abortion, unwanted pregnancy, cesarean delivery, maternal alcohol and tobacco exposure during pregnancy, epilepsy, and head trauma were significantly more among ADHD children than control group. Accordingly, avoiding alcohol, tobacco, and unnecessary X-ray during pregnancy and elective cesarean delivery are recommended as well as feeding by breast milk instead of formula. However, it must be taken into consideration that alcohol and tobacco exposure, previous abortion, and unwanted pregnancy may be results of maternal and paternal ADHD.

## Figures and Tables

**Table 1:** Maternal, pregnancy, and neonatal risk factors for ADHD among case and control subjects.

Proposed risk factor	Group	Number (%)	OR (95% CI)	*P* value
Trauma to abdomen in pregnancy	ADHD	7 (3.4%)	4 (0.000–1000)	0.039
	Control	1 (0.5%)		
X-ray exposure	ADHD	2 (1%)		NS*
	Control	1 (0.05%)		
Vaginal bleeding	ADHD	8 (3.9%)		NS
	Control	11 (5.6%)		
Infectious disease during pregnancy	ADHD	7 (3.4%)		NS
	Control	5 (2.5%)		
Cigarette and alcohol consumption	ADHD	25 (12.1%)		<0.0001
	Control	4 (2%)		
Preeclampsia	ADHD	20 (9.7%)		0.009
	Control	6 (3%)		
Dysentery	ADHD	8 (3.9%)		NS
	Control	11 (5.6%)		
Cesarean section	ADHD	107 (51.7%)		0.019
	Control	81 (40.9%)		
Unwanted pregnancy	ADHD	29 (14%)	4.2 (0.000–1000)	<0.0001
	Control	5 (2.5%)		
Previous abortion	ADHD	36 (17.4%)	24 (0.594–977.082)	<0.0001
	Control	11 (5.6%)		
Somatic disease at pregnancy	ADHD	14 (6.8%)		0.007
	Control	3 (1.5%)		
Psychiatric disease at pregnancy	ADHD	17 (8.2%)		0.004
	Control	4 (2%)		
Formula feeding	ADHD	75 (36.6%)		<0.0001
	Control	39 (24.9%)		

*NS: not significant.

**Table 2:** Chi-square test for parental psychiatric disorders and their familial relation among case and control groups.

	Group	Number (%)	OR (95% CI)	*P* value
Related parents	ADHD	51 (24.6%)		0.012
	Control	70 (35.4%)		
Maternal psychiatric disorder	ADHD	46 (22.2%)		<0.0001
	Control	11 (5.6%)		
Paternal psychiatric disorder	ADHD	52 (25.2%)	8.7 (0.71–106.3)	<0.0001
	Control	19 (9.6%)		

**Table 3:** Neonatal and childhood related risk factors for ADHD among case and control groups.

	Group	Number (%)	OR (95% CI)	*P* value
Asthma	ADHD	20 (9.7%)		NS
	Control	17 (8.6%)		
Epilepsy	ADHD	13 (6.3%)		0.001
	Control	1 (0.5%)		
Dysentery	ADHD	5 (2.4%)		NS
	Control	3 (1.5%)		
Childhood head trauma	ADHD	44 (21.3%)	4.4 (0.000–1000)	<0.0001
	Control	2 (1%)		
Thyroid disease	ADHD	2 (1%)		NS
	Control	0 (0%)		
Hyperbilirubinemia	ADHD	101 (48.8%)		NS
	Control	81 (40.9%)		
Low birth weight	ADHD	16 (7.8%)		NS
	Control	24 (12.1%)		
